# Use of confocal laser endomicroscopy with a fluorescently labeled fatty acid to diagnose colorectal neoplasms

**DOI:** 10.18632/oncotarget.19515

**Published:** 2017-07-24

**Authors:** Feihong Deng, Yuan Fang, Zhiyong Shen, Wei Gong, Tao Liu, Jing Wen, Wanling Zhang, Xianjun Zhu, Hui Zhong, Tong Wang, Fachao Zhi, Biao Nie

**Affiliations:** ^1^ Department of Gastroenterology, Nanfang Hospital, Southern Medical University, Guangzhou, Guangdong Province, 510515 China; ^2^ Department of General Surgery, Nanfang Hospital, Southern Medical University, Guangzhou, Guangdong Province, 510515 China; ^3^ Department of Gastroenterology, First Affiliated Hospital of Jinan University, Jinan University, Guangzhou, Guangdong Province, 510630 China; ^4^ Key Laboratory of Functional Protein Research of Guangdong Higher Education Institutes, Institute of Life and Health Engineering, College of Life Science and Technology, Jinan University, Guangzhou, Guangdong Province, 510632 China

**Keywords:** fatty acid metabolism, colorectal neoplasm, confocal laser endomicroscopy, fluorescent agents, de novo FA synthesis

## Abstract

Endoscopic treatment for early colorectal cancer closely correlates with patient prognosis. However, endoscopic differentiation between carcinomas and non-neoplastic lesions remains difficult. Here, we topically stained colorectal neoplasms with a fatty acid analogue (BODIPY-FA) and quantified the fluorescent signals using confocal laser endomicroscopy (CLE) and fluorescence microscopy. We also analyzed protein expression in colorectal cancer tissues. We found that expression of fatty acid synthase was elevated, while the expression of fatty acid transporters was reduced in colorectal cancer. In colorectal cancer mouse models and patients, the BODIPY-FA signals were higher in normal epithelia than in carcinomas or colonic intraepithelial neoplasias. BODIPY-FA staining revealed both the arrangement of intestinal glands and the intracellular structures under CLE screening. In a double-blind trial, CLE images stained with BODIPY-FA exhibited greater consistency (κ = 0.68) and overall validity (74.65%) than those stained using intravenous fluorescein sodium (κ = 0.43, 55.88%) when the results were compared with histological diagnoses. These findings suggest that topical use of BODIPY-FA with CLE is a promising imaging approach for early colorectal neoplasm screening.

## INTRODUCTION

Cancer cell metabolism differs significantly from normal cell metabolism. Most proliferating cancer cells take up more glucose and produce more lactate via the glycolytic pathway than normal cells, even when oxygen is abundant [1–5]. This so-called ‘Warburg effect’ has been intensively studied and generally applied in clinical positron emission tomography–computed tomography [6, 7]. In addition, endogenous fatty acid (FA) synthesis (also called *de novo* FA synthesis) is specifically enhanced in cancer cells, irrespective of the exogenous lipid supply [8–11]. This process is another important metabolic hallmark of cancer [12–14]. *De novo* FA synthesis generates long-chain fatty acids (LCFAs) from acetyl-CoA, and accounts for the synthesis of almost all esterified FAs and more than 93% of triglycerides to meet the biosynthetic demands of tumors [15]. However, the practical applications of this phenomenon have not been fully explored.

As colorectal cancer (CRC) is the third-most-common cancer in males and the second-most-common cancer in females worldwide [16], early CRC detection is crucial for improving the quality of life and the cure rate for patients. Colonoscopy has been regarded as the gold standard for screening people at high risk for CRC. Multiple novel endoscopic technologies have recently been investigated, including narrow-band imaging (NBI), Fujinon intelligent chromoendoscopy (FICE), magnifying chromoendoscopy (MCE) and confocal laser endomicroscopy (CLE) [17, 18]. CLE integrates experimental confocal microscopy with conventional optical endoscopy, magnifying regions of interest (ROIs) 1000-fold at the cellular and sub-cellular levels and allowing real-time *in vivo* histology in the clinical diagnosis of colorectal neoplasms [19, 20]. Although fluorescently labeled antibodies and peptides have been tested with CLE [21–24], labeled FAs have never been used in trials to evaluate colorectal neoplasms specifically on a metabolic level.

The fluorescently labeled FA analogue BODIPY-FA (4,4-difluoro-5-methyl-4-bora-3a,4a diaza-s-indacene-3-dodecanoic acid) is activated by argon-ion laser excitation at 488 nm, and has generally been applied for experimental research in lipid trafficking [25]. In this study, we found that BODIPY-FA was a promising biomarker for tumor imaging, due to the reprogramming of FA metabolism in CRC cells. Furthermore, upon its absorption, BODIPY-FA was enriched in the cytoplasm, and created contrast that allowed negative nuclear visualization. Thus, BODIPY-FA provided histological imaging similar to hematoxylin and eosin (H&E) staining, making it superior to other newly developed contrast agents.

## RESULTS

### FA metabolism differed between normal colorectal and CRC samples

**Figure 1:**
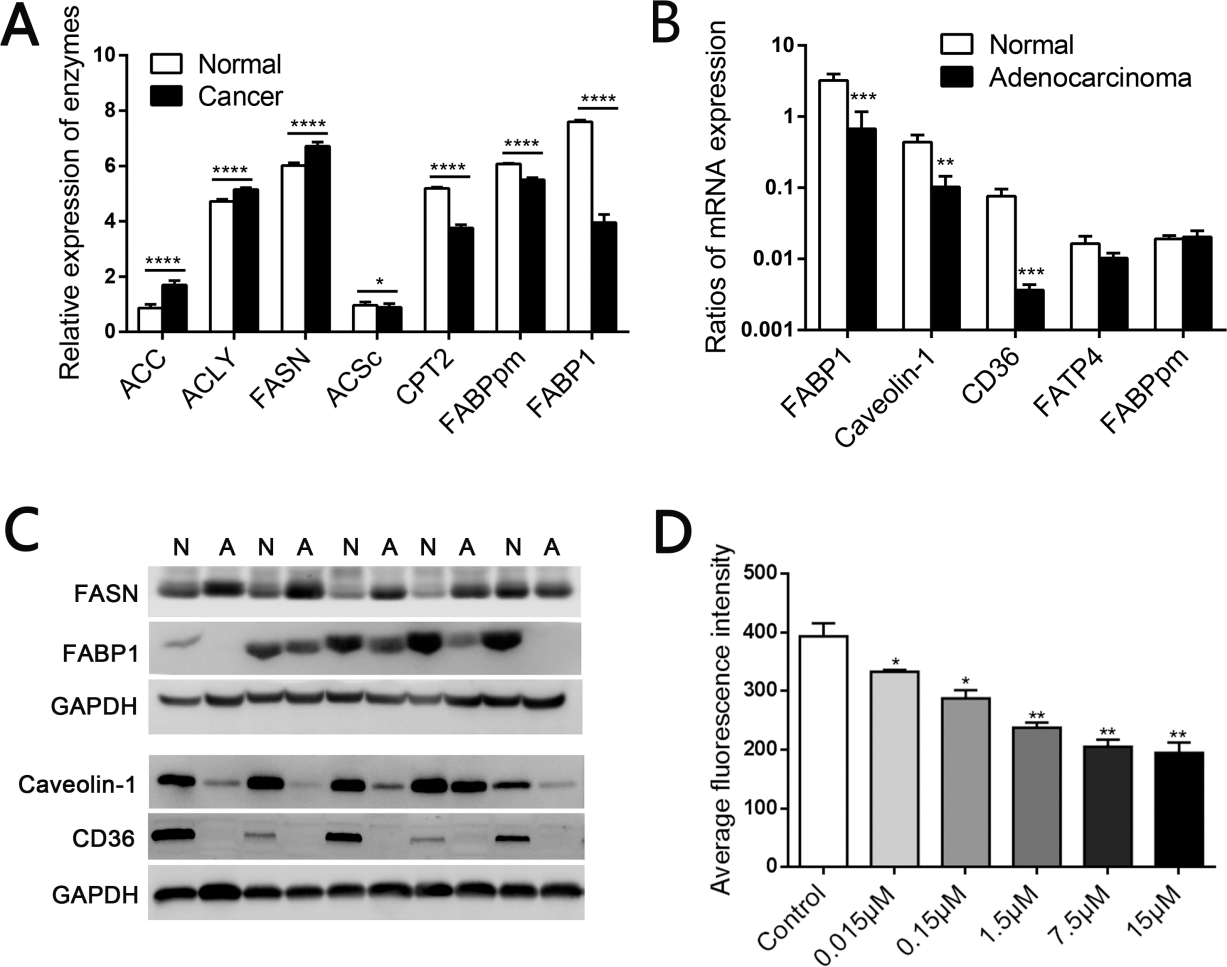
Elevated *de novo* fatty acid synthesis combined with reduced fatty acid oxidation in human CRC (**A**) Mass spectrometry-based proteomic analysis of 95 colorectal cancer samples from the Clinical Proteomic Tumor Analysis Consortium. ACC, acetyl-CoA carboxylase; ACLY, ATP citrate lyase; FASN, fatty acid synthase; ACSc, acetyl-CoA synthetase complex; CPT2, carnitine palmitoyl transferase 2. (**B**) The mRNA levels of relevant LCFA transporters including *FABP1*, *Caveolin-1* and *CD36* were lower in CRC tissues than in matched normal mucosae. (**C**) The protein levels of FASN, FABP1, Caveolin-1 and CD36 in CRC tissues and matched normal mucosae. N, Normal; A, Adenocarcinoma. (**D**) Competition in the fluorescent signal after various concentrations of C18:0 were added to 2 μM BODIPY-FA medium on SW480 cells. *P < 0.01, **P < 0.001, ***P < 0.001, ****P < 0.0001. Bars indicate the SEMs.

Analysis of data from the Clinical Proteomic Tumor Analysis Consortium indicated that most enzymes involved in *de novo* FA synthesis, including FASN, acetyl-CoA carboxylase (ACC) and ATP citrate lyase (ACLY), were expressed at significantly higher levels in CRC tissues than in normal colorectal tissues. In addition, the acetyl-CoA synthetase complex (ACSc) and carnitine palmitoyl transferase 2 (CPT2) were downregulated in CRC tissues, suggesting that FA oxidation is impaired in colonic carcinoma (Figure 1A and Supplementary Figure 1, detailed in Supplementary Table 4). More importantly, concomitant with the enhancement of endogenous FA synthesis, the expression of membranal transporters of LCFAs, such as plasma membrane FABP (FABPpm) and FABP1, decreased sharply in CRC (Figure 1A and Supplementary Figure 1). Western blotting revealed that FASN protein expression was elevated in CRC tissues, while real-time PCR and Western blotting indicated that FABP1, CD36 and Caveolin-1 mRNA and protein levels were reduced in CRC (Figure 1B, 1C), potentially due to the reduced availability of exogenous lipids for the tumor.

LCFA transport begins with the uptake of circulating non-esterified FAs (derived from triglycerides) by membranal transporters. Previously, FA uptake studies relied on radiolabeled lipids, and thus lacked real-time dynamic monitoring and were costly [32]. BODIPY-FA is a fluorescently labeled FA consisting of a 12-carbon saturated FA chain connected to the fluorophore BODIPY. BODIPY-FA biologically resembles an 18-carbon FA, and is reported to be a suitable alternative to radiolabeled FAs [33]. Stearic acid (C18:0) is a dominant member of the non-esterified LCFA family, and is the main source of FA oxidation for cellular energy after its uptake by lipid transporters [34]. Through FACS analysis, we found that stearic acid could effectively compete with the BODIPY-FA signal in SW480 cells (Figure 1D), confirming that BODIPY-FA is a C18:0 analogue. Thus, BODIPY-FA could be a tool for monitoring lipid transport, and could reflect the natural uptake of circulating non-esterified LCFAs *in vivo*.

### BODIPY-FA uptake was reduced in human CRC cells

**Figure 2:**
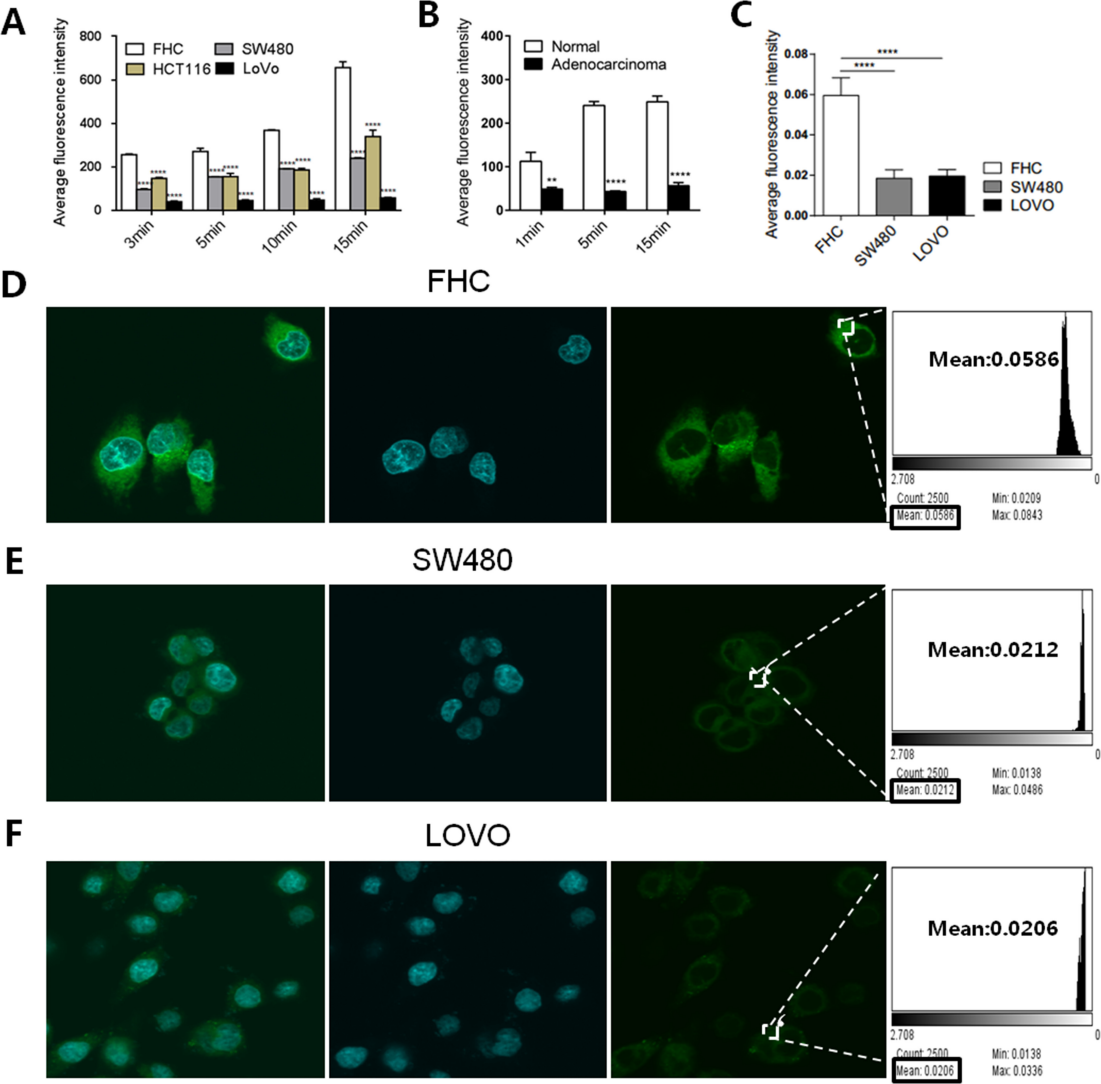
BODIPY-FA absorption by CRC cell lines and normal FHC cells (**A**) Adherent cell uptake of BODIPY-FA at the indicated time points, as determined with FACS analysis. (**B**) BODIPY-FA uptake by suspensions of primary colonic normal and adenocarcinoma cells, as determined with FACS analysis. (**C**) The average fluorescence intensity of BODIPY-FA in colonic cells after CLSM screening. (**D**, **E**, **F**) CLSM imaging of adherent cell uptake of BODIPY-FA within 15 minutes, to confirm that FHC cells absorbed more BODIPY-FA than the CRC cell lines. *P < 0.05, **P < 0.01, ****P < 0.0001. Bars indicate the SEMs.

Based on the downregulation of FA transporters in human CRC tissues, we hypothesized that exogenous FA uptake is limited in cancer. To verify this hypothesis, we used BODIPY-FA to examine the differences in FA metabolism between CRC cells and normal colonic cells *in vitro*. FACS analysis demonstrated that BODIPY-FA accumulated in adherent cells within 15 minutes, and that the fluorescent signal was greater in a normal colonic cell line (FHC) than in CRC cell lines (SW480, HCT116, and LOVO) (*P* < 0.0001, Figure 2A). FACS analysis of three pairs of fresh cancerous and matched normal colonic tissues revealed the same trend in the BODIPY-FA signal, and the absorption of normal primary colonic epithelial cells plateaued within five minutes (Figure 2B).

Next, we determined the ability of confocal laser microscopy to locate and discriminate BODIPY-FA staining. A stronger cellular signal was observed for FHC cells than for CRC cells (SW480, LOVO) (*P* < 0.0001, Figure 2C). In addition, the specific BODIPY-FA signal was cytoplasmic and perinuclear (Figure 2D–2F). These data indicated that LCFA uptake was reduced in CRC cells, and that limited exogenous FA usage may be an intrinsic signature of tumor lipid metabolism.

### Fluorescence microscopy revealed reduced BODIPY-FA uptake by colonic neoplasms in mouse models *in vivo*

**Figure 3:**
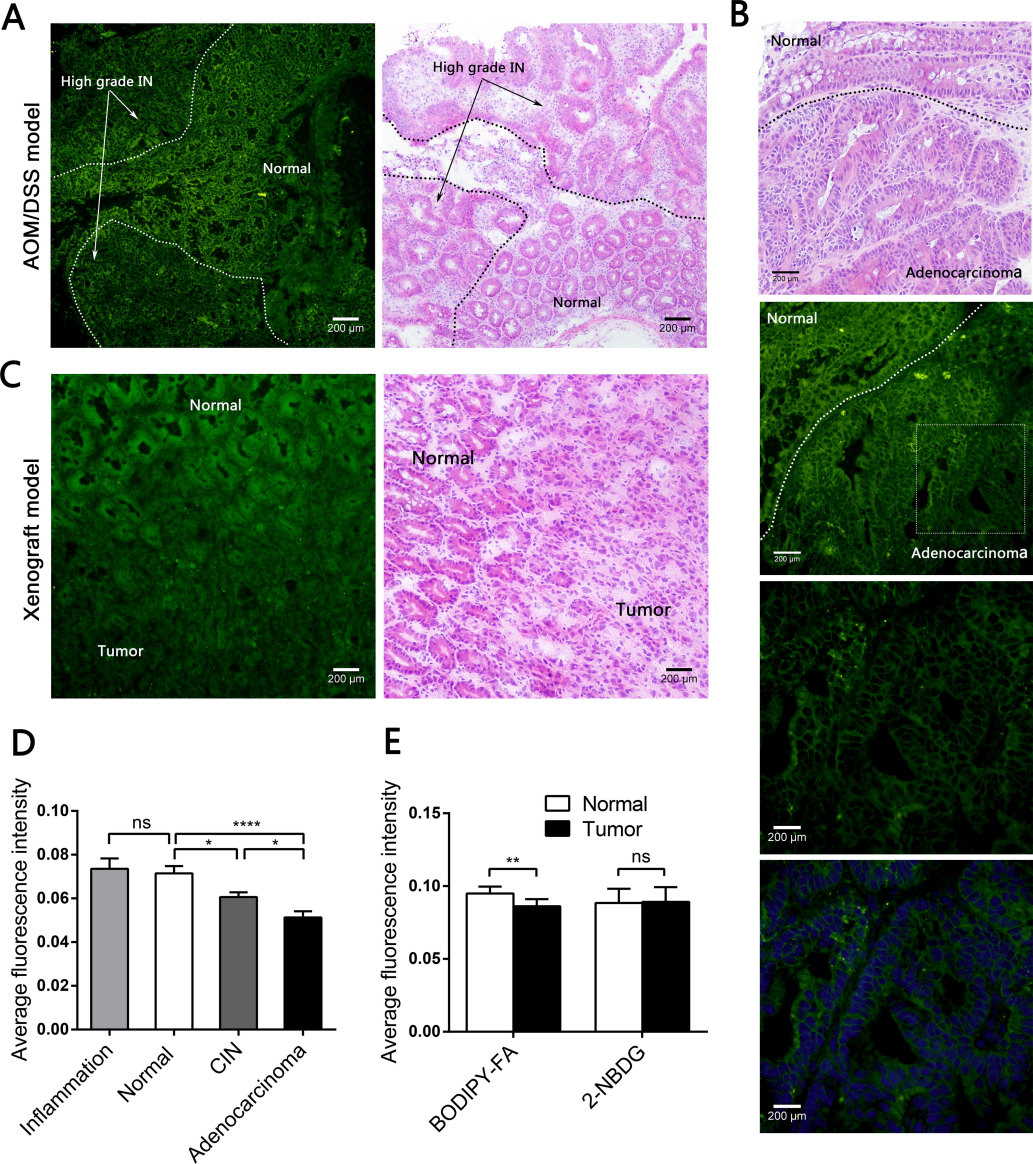
Fluorescence microscopy imaging after topical application of BODIPY-FA to the mucosae of mouse models, and the corresponding H&E images (**A**) BODIPY-FA signal at the transition between the normal mucosa and high-grade IN in an AOM/DSS-induced mouse at the end of three cycles. Images were obtained at 100×. (**B**) BODIPY-FA staining of the transition between normal and adenocarcinoma mucosae in an AOM/DSS-induced mouse, as observed at 200×; the details of the adenocarcinoma and the corresponding DAPI nuclear counterstaining are presented at 400×. (**C**) Imaging of a LOVO xenograft mouse at the transition between normal and tumorous tissue, four weeks after implantation, shown at 200×. (**D**) The average fluorescence intensity of BODIPY-FA in different tissue types of AOM/DSS-induced mice. (**E**) The average fluorescence intensities of BODIPY-FA and 2-NBDG in normal and tumorous tissues from xenograft mice. *P < 0.05, **P < 0.01, ****P < 0.0001; ns, not significant. Scale bars: 200 μm. Bars indicate the SEMs.

Next, we topically administered fluorescent agents to both AOM/DSS-induced and xenograft models, and analyzed the cytoplasmic fluorescence intensity under fluorescence microscopy. When we analyzed the colitis-related carcinomas produced in the AOM/DSS model, we found a stepwise decrease in the BODIPY-FA signal, which was strong in non-neoplastic samples, lower in CIN samples, and even lower in adenocarcinoma samples (Figure 3A, 3B, Supplementary Figure 3A). In addition, the BODIPY-FA signals from adenocarcinoma and CIN samples were approximately 39.3% (*P* < 0.0001) and 17.7% (*P* = 0.0117) lower, respectively, than in normal epithelia (Figure 3D). The BODIPY-FA signals from orthotopically implanted tumors of xenograft mice were approximately 10.2% lower than those of paired normal mucosae (*P* = 0.0017, Figure 3C, 3E).

Based on this difference, we used the deoxyglucose analogue 2-NBDG as a control, in which 2-deoxyglucose is linked to the fluorophore N-(7-nitrobenz-2-oxa-1,3-diaxol-4-yl)amino (NBD). The 2-NBDG signals were approximately 20% greater in neoplastic mucosae than in normal mucosae from AOM/DSS-induced mice (*P* = 0.0119, Supplementary Figure 2A, 2C). The 2-NBDG absorption was also higher in inflamed tissues than in normal mucosae (*P* = 0.0209, Supplementary Figure 3B). However, there were no differences between the normal tissues and the tumors in the xenograft model after 2-NBDG staining (Supplementary Figure 2B). All fluorescent agent signals were greater than the background autofluorescence.

### CLE revealed reduced BODIPY-FA uptake by colonic neoplasms in mouse models *in vivo*

**Figure 4:**
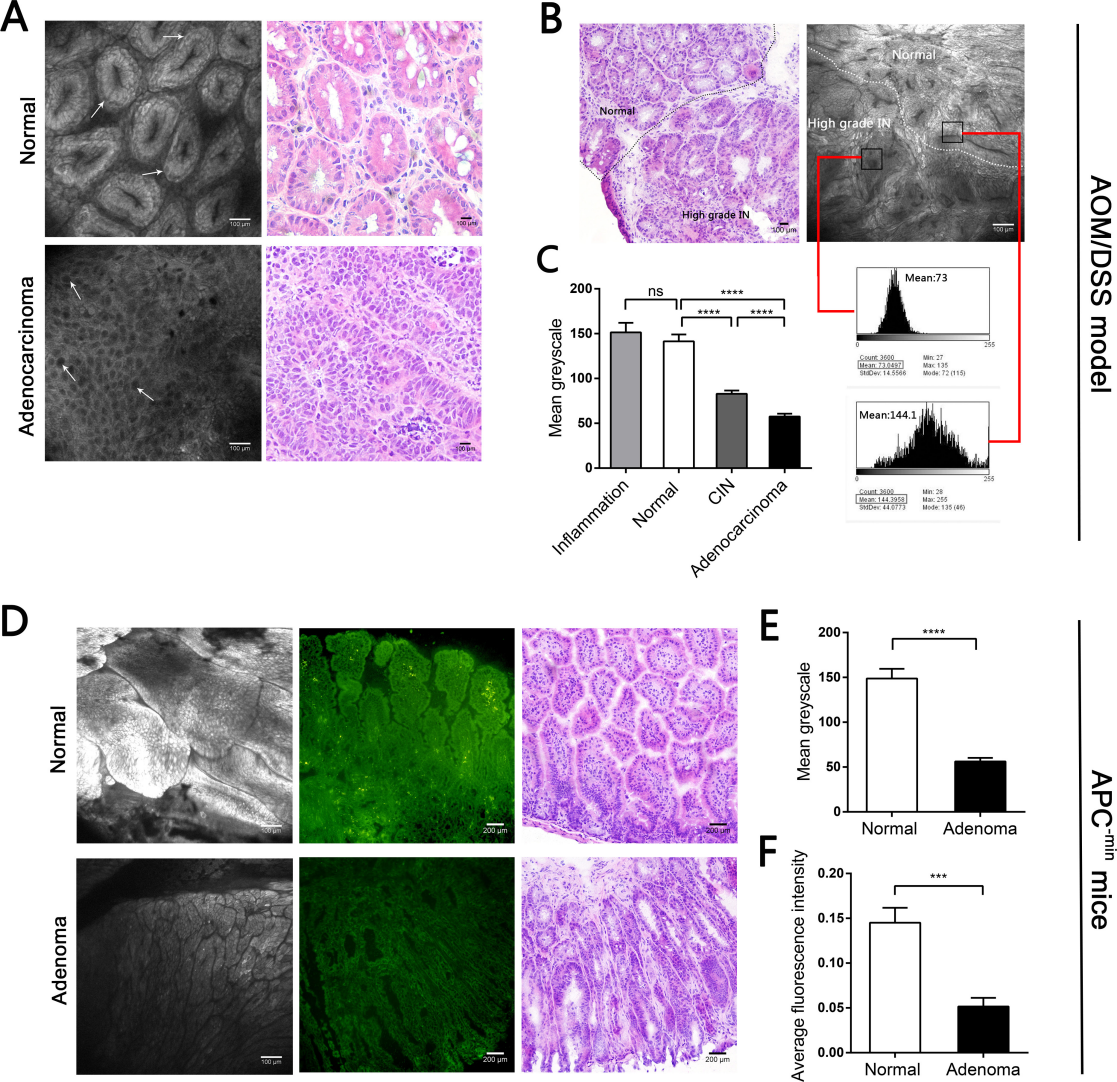
CLE images following topical application BODIPY-FA to the mucosae of mouse models, and the corresponding H&E images (**A**) BODIPY-FA expression in normal and adenocarcinoma mucosae from the AOM/DSS mouse model. Arrows indicate the morphology of the nuclei. (**B**) Imaging of BODIPY-FA at the transition between the normal mucosa and high-grade IN in an AOM/DSS-induced mouse, and the corresponding H&E images. Histograms display the greyscale values of the normal and high-grade IN regions. (**C**) The mean greyscale values of the BODIPY-FA signals in different tissue types of AOM/DSS-induced mice. (**D**) BODIPY-FA staining in normal and adenoma sites of APC^-min^ mice, as observed with CLE, and the corresponding fluorescence imaging. (**E**) The mean greyscale values of the BODIPY-FA signals in APC^-min^ mice. (**F**) The average intensity of BODIPY-FA in APC^-min^ mice. ***P < 0.001, ****P < 0.0001; ns, not significant. Bars indicate the SEMs.

**Figure 5:**
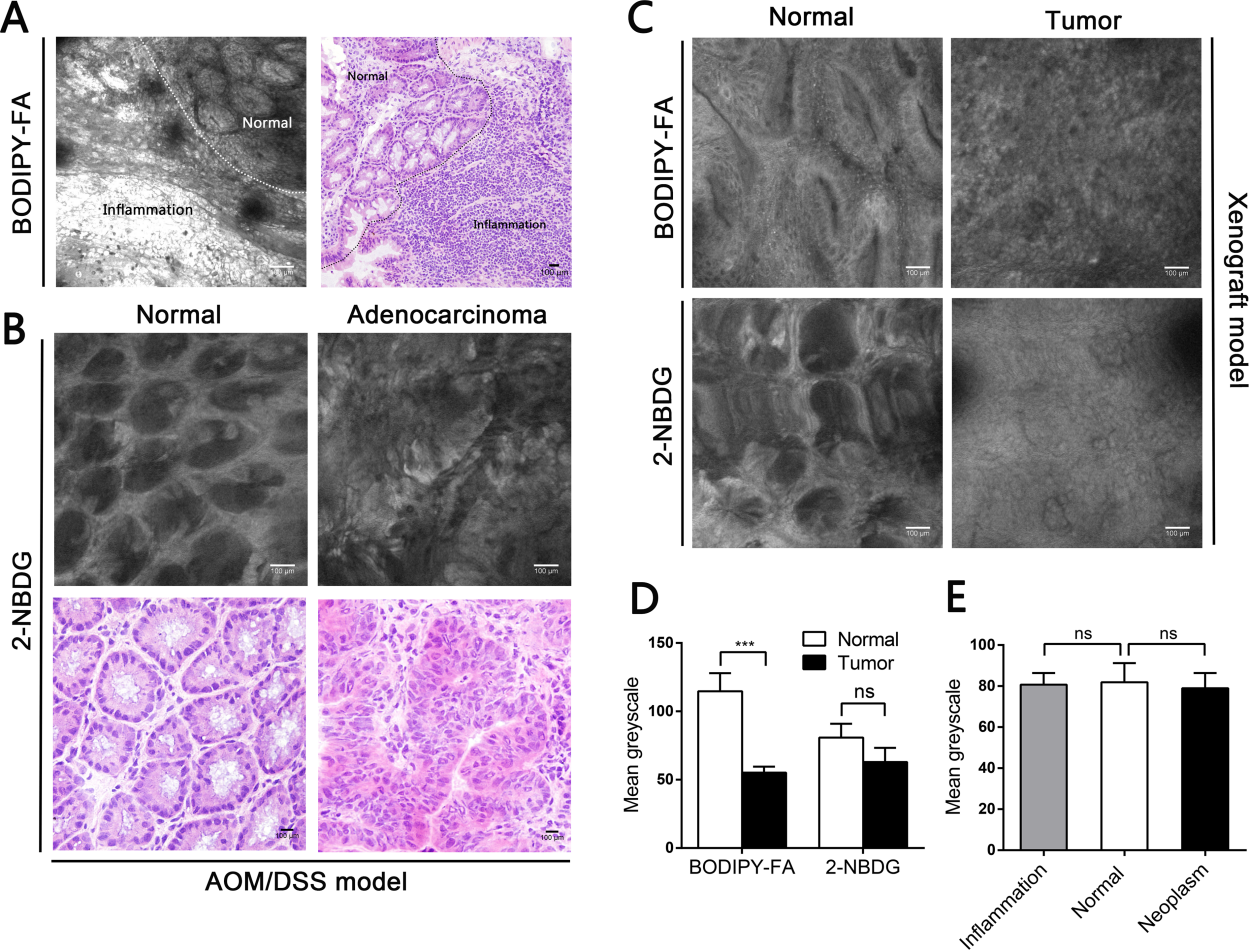
CLE images following topical application of fluorescent agents to the mucosae of mouse models, and corresponding H&E images (**A**) Imaging of BODIPY-FA signals at the transition between inflammation and the normal mucosa of an AOM/DSS-induced mouse at the end of one cycle. (**B**) 2-NBDG staining in normal and adenocarcinoma mucosae. (**C**) BODIPY-FA and 2-NBDG staining in normal and tumorous sites of LOVO xenograft mice. (**D**) The mean greyscale values of the fluorescent signals in normal and tumorous regions of LOVO xenograft mice. (**E**) The mean greyscale values of the 2-NBDG signals in different tissue types of AOM/DSS-induced mice. ***P < 0.001; ns, not significant. Bars indicate the SEMs.

Because of the BODIPY-FA signal differences observed with fluorescence microscopy, we then performed CLE with topically applied fluorescent agents to explore the uptake differences *in vivo*. In AOM/DSS-induced mice, the BODIPY-FA signals declined progressively from inflamed to normal to CIN to adenocarcinoma tissues (Figure 4A, 4B and Figure 5A). The mean greyscale values of the normal mucosae were 2.5 and 1.7 times greater than those of adenocarcinomas and CIN, respectively (Figure 4C). In parallel, the BODIPY-FA signal was 2.1-fold higher in normal tissues than in colonic orthotopic tumors (Figure 5C, 5D). For APC^-min^ mice, the BOPIDY-C12 signal was significantly higher in normal mucosae than in adenomas, according to CLE (*P* < 0.0001) and fluorescence microscopy (*P* = 0.0001) (Figure 4D–4F). Furthermore, changes in cellular structures, such as the disorganized cellular arrangement and cellular atypia in the neoplastic lesions, were more well-defined under CLE with BODIPY-FA staining than with other contrast agents.

In contrast, CLE following topical administration of 2-NBDG did not facilitate clear outlining of the glands, let alone cytoplasmic imaging, and no fluorescent difference between neoplastic and non-neoplastic tissues was observed in the mouse models (Figure 5B, 5E). For acriflavine staining, the nuclei within the ROIs were all displayed (Supplementary Figure 4A, 4B). No background autofluorescence was observed in normal or malignant tissues.

### CLE revealed reduced BODIPY-FA uptake in colonic neoplasms in patients

**Figure 6:**
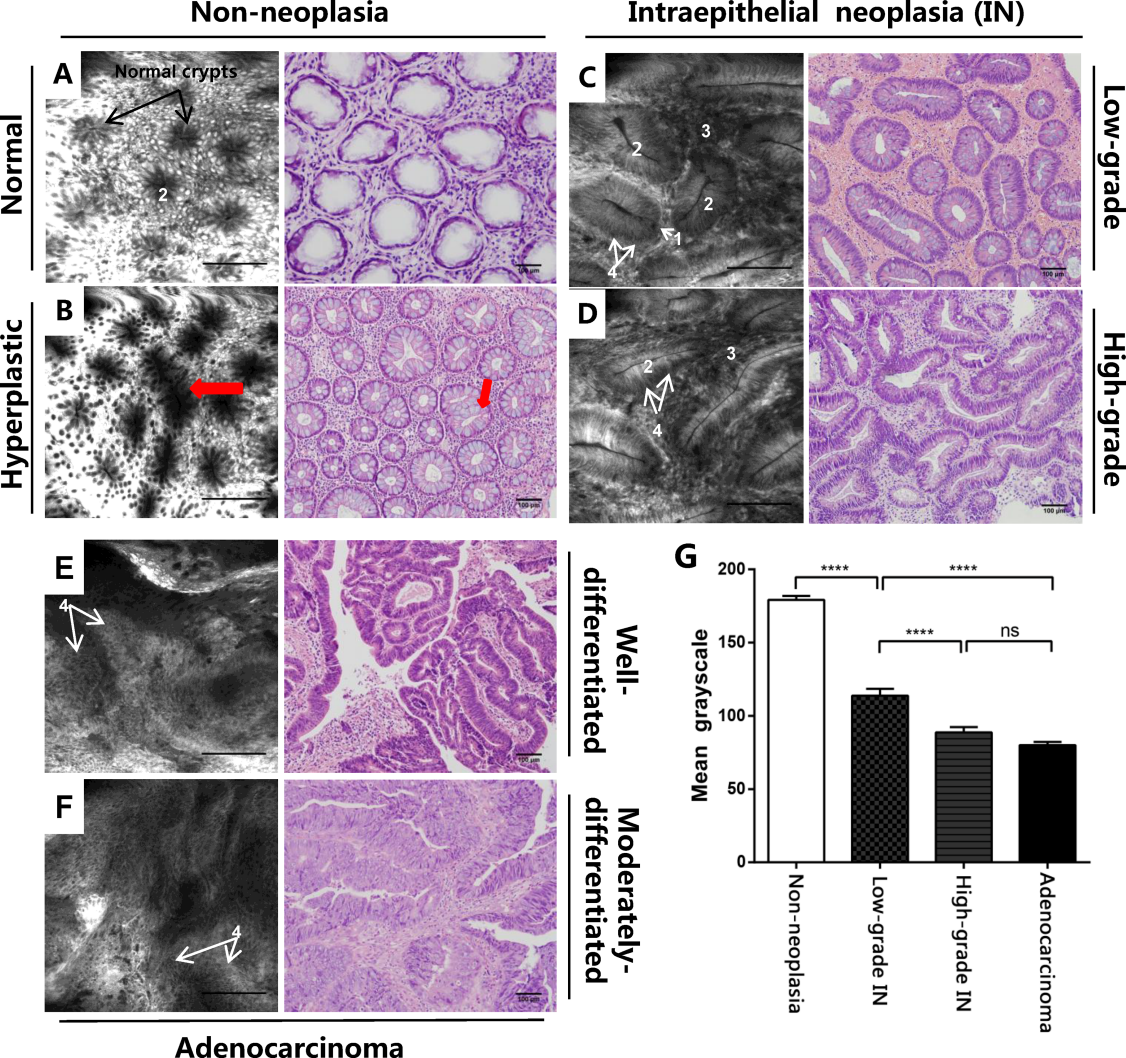
CLE imaging of patient samples and the corresponding H&E images (**A**) CLE imaging of the normal colonic mucosa and (**B**) typical star-shaped crypt openings and fused regularly shaped crypts (arrowheads) at the regenerative location. (**C**) Images of mucosal low-grade IN display elongated crypts with reduced goblet cells. (**D**) Images of high-grade IN display branch-like or extremely stretched crypts with lateral fusion of irregular glands and an elevated nucleus-to-cytoplasm ratio. (**E**) A well-differentiated adenocarcinoma exhibits enlarged black nuclei, with an enhanced nucleus-to-cytoplasm ratio. (**F**) Images of a moderately-differentiated adenocarcinoma exhibit extremely disorganized or absent glands. (**G**) The mean greyscale values of BODIPY-FA signals in different tissue types from patient samples. Legend: 1. Goblet cells, 2. Crypt lumen, 3. Lamina propria, 4. Nucleus. ****P < 0.0001; ns, not significant. Bars indicate the SEMs.

We further used BODIPY-FA with CLE to explore lipid uptake in human samples. The mean greyscale values of all distinguishable confocal images (149 non-neoplasm images, 103 low-grade IN images, 132 high-grade IN images, and 124 adenocarcinoma images) of patients were analyzed under the same excitation brightness control. Images with obvious impurities on the tissue surface were eliminated from the assessment. The BODIPY-FA signals of non-neoplastic sites were almost 1.6-, 2- and 2.2-fold higher than those of low-grade IN, high-grade IN and cancerous sites, respectively, but the signals of high-grade IN and adenocarcinoma sites did not differ significantly from one another (Figure 6G).

### Staining characteristics of BODIPY-FA and classification of colorectal neoplasms by CLE

We also analyzed the characteristics of BODIPY-FA staining with CLE. At normal sites, black nuclei on the basal side and bright cytoplasmic staining facilitated the visualization of regularly arranged crypts, which were surrounded by epithelial cells and goblet cells. With regard to hyperplastic polyps, focal aggregations of regularly shaped crypts or typical star-shaped luminal crypt openings were visible, and the number of goblet cells was normal or slightly reduced (Figure 6A, 6B). BODIPY-FA staining on adenocarcinomas was greatly altered, revealing many atypical cells with large and irregularly shaped nuclei (Figure 6E, 6F).

Next, we evaluated the potential use of BODIPY-FA with CLE for the early diagnosis of neoplasms. In low-grade IN, BODIPY-FA-stained lesions exhibited slightly atypical or elongated glands with a single layer of nuclei on the basal side. Moreover, the nucleus-to-cytoplasm ratio was almost normal, in spite of the slightly reduced number of goblet cells. Strikingly, for high-grade IN, ROIs displayed lateral fusion of destroyed crypts, extremely elongated and branch-like crypts, and an increased nucleus-to-cytoplasm ratio. In addition, goblet cells were either reduced in number or completely absent (Figure 6C, 6D).

### Comparison between CLE diagnosis and clinical histological findings

Finally, we performed a clinical comparison of CLE diagnosis and histological assessments. The inter-observer agreement was greater for BODIPY-FA staining (κ = 0.72, *P* < 0.001) than for fluorescein sodium (κ = 0.61, *P* < 0.001). The overall validity of CLE versus H&E diagnosis was 74.65% with BODIPY-FA and 55.88% with fluorescein sodium. In addition, there was good diagnostic consistency (κ = 0.68, *P* < 0.001) between BODIPY-FA images and H&E diagnosis. However, although the diagnostic difference was slight (chi-square = 1.10, *P* = 0.9540), the consistency with the histological diagnosis was worse for fluorescein sodium than for BODIPY-FA (κ = 0.43, *P* < 0.001). These results are detailed in Supplementary Tables 2 and 3, indicating that CLE with BODIPY-FA staining had greater consistency and overall validity than CLE with intravenous fluorescein sodium.

## DISCUSSION

Endoscopic treatment for CRC at the early stage can effectively improve the cure rate for patients. In this study, we found that lipid metabolism was aberrant in CRC, and that the uptake of the fluorescently labeled FA BODIPY-FA was reduced in CRC tissues from both mouse models and human patients. BODIPY-FA staining under CLE effectively differentiated non-neoplastic tissues, colonic IN and carcinomas, as it created a strong fluorescent contrast and allowed clear visualization of cellular structures. BODIPY-FA with CLE enabled real-time cellular and sub-cellular imaging of ROIs, and defined a much more accurate boundary between neoplasm and non-neoplasm than existing methods; thus, this technique may facilitate early CRC identification for further clinical treatments.

Multiple novel endoscopic technologies have been investigated for early CRC identification, such as NBI, FICE, MCE and CLE. NBI can differentiate neoplasms from non-neoplasms according to surface microvessel changes, but poorly detects depressed-type lesions [35]. MCE with crystal violet staining has a diagnostic accuracy of 85–88% based on surface pit pattern identification [36]. However, disappointing misdiagnoses and missed diagnoses still occur, due to the variety of superficial lesions and the limited role of forceps biopsy; in fact, the disagreement between histopathology and resected biopsy tissues has been reported to be approximately 40% [37]. In contrast, with its 1000× magnifying visualization, CLE has been shown to have 93.9% sensitivity and 95.9% specificity for colonic adenoma detection [38].

CLE diagnosis has relied exclusively on fluorescent staining agents such as fluorescein sodium and acriflavine thus far. BODIPY-FA is a C18:0 analogue, and can distinctly highlight the structure of the cell nucleus in mouse models when it is applied topically. Although systemic agents such as fluorescein sodium have the advantage of reduced immunogenicity, their localization in blood vessels prevents them from displaying the morphology of the intracellular architecture. Topical agents have been used preferentially in clinical settings [39, 40], since they allow real-time imaging of ROIs and can be applied easily during CLE. In our trial, due to its low molecular weight and hydrophobic features, BODIPY-FA rapidly entered the cytoplasm (within five minutes) and accumulated stably in the cytoplasm. Given its rapid and relatively stable imaging of ROIs, CLE screening with BODIPY-FA is more convenient and efficient than CLE with fluorescein sodium.

In addition to rapid cellular imaging, in our study, BODIPY-FA with CLE effectively visualized enlarged glands with different arrangements and, more importantly, distinguished between normal and aberrant nucleus-to-cytoplasm ratios in both mouse models and human tissues. In contrast, 2-NBDG failed to visualize nuclei or glands under CLE. Furthermore, the overall prediction consistency and accuracy were much greater for CLE with BODIPY-FA than with fluorescein sodium. Thus, malignant lesions displayed weak BODIPY-FA signals, accompanied by increased nucleus-to-cytoplasm ratios under CLE. In addition, as the elevated BODIPY-FA signals in inflamed sites were parallel to those in normal mucosae, BODIPY-FA imaging with CLE also facilitated the detection of colitis-associated carcinoma, thus assisting in the diagnosis of endoscopic depressed-type lesions. Overall, BODIPY-FA staining under CLE exploited differences in lipid metabolism and enhanced cellular visualization, thereby improving the diagnostic efficiency of CLE screening during CRC identification.

De novo FA synthesis is a common metabolic characteristic of cancer, and has been studied in various human cancers, including breast, colorectal, lung, prostate and liver cancer [41, 42]. This process is controlled by rate-limiting enzymes such as FASN and ACC, and generates FAs de novo from acetyl-CoA. Inhibitors of FASN (C75 or TVB-3166) or ACC (ND-646) have been shown to effectively suppress the growth and viability of cancer cells, demonstrating the importance of endogenous FA synthesis in oncology [43, 44]. Strikingly, exogenous FAs, in addition to being the main source of cellular energy, generally compete with *de novo* FA synthesis [45, 46]. In a previous study, growing cells in lipid-depleted medium clearly enhanced the effects of ND-646 by placing a heavier burden on endogenous FA synthesis [44]. On the other hand, the addition of palmitate fully restored cancer cell viability after the inhibition of FASN [43]. In our study, we found that FASN expression was increased in human CRC, while the expression of membranal transporters of LCFAs such as FABP1, CD36 and Caveolin-1 was reduced. The reduced expression of lipid transporters may further impair extracellular lipid uptake in CRC tissues relative to normal colonic mucosae. Further BODIPY-FA detection *in vitro* and *in vivo* also verified these findings. Thus, we propose that limited exogenous lipid usage directly manifests itself through the downregulation of LCFA transporters, and could be a response to increased *de novo* FA synthesis in CRC tissues. Further molecular research could focus on this interesting subject.

In summary, abnormal lipid metabolism in cancer cells caused distinct uptake patterns of the fluorescently labeled FA BODIPY-FA in non-neoplastic mucosae and neoplastic tissues, providing a novel approach for early CRC detection. This method specifically identified malignant sites with “dark areas” and allowed well-defined intracellular imaging under CLE, similar to “real-time histochemistry,” which enhanced the detection of suspicious lesions and enabled relatively reliable diagnoses. This method was also beneficial for the detection of inflammatory bowel disease-associated carcinoma, and could be a reliable screening tool for at-risk patients. We speculate that reduced exogenous FA uptake may be an inherent neoplastic characteristic that is representative of most other tumors. This research will open a new door to tracing pre-neoplastic or neoplastic lesions in scientific research and clinical practice.

## MATERIALS AND METHODS

### Cell culture and fluorescence-activated cell sorting (FACS)-based BODIPY-FA uptake assays

All cells were obtained from the American Type Culture Collection (Maryland, USA). Different CRC cell lines were cultured in Dulbecco's Modified Eagle Medium (10% fetal calf serum, 1% glutamine, 1% streptomycin /penicillin) at 37°C and 5% CO_2_. When cells reached 60–80% confluency, they were plated in 12-well plates covered with aluminum foil at a density of 500,000 cells/well and incubated overnight for adherence. BODIPY-FA (D3823, Invitrogen, Carlsbad, CA, USA) was dissolved in dimethyl sulfoxide as a stock solution [25], and the working solution was diluted with 1× phosphate-buffered saline (PBS) and 0.1% FA-free bovine serum albumin. Different cell lines were cultivated in 2 μM BODIPY-FA for 1–15 minutes. For fluorescent competition with stearic acid, SW480 cells were cultivated in various concentrations of a BODIPY-FA-stearic acid mixture for 15 minutes. Then, the cells were rinsed and harvested immediately for FACS analysis.

We also isolated primary colonic epithelial cells from cancerous and matched normal tissue samples, as described previously [26], after obtaining informed consent from the patients. Details on the patients are given in Supplementary Table 1.3.

For cellular staining with BODIPY-FA, FHC and CRC cell lines were cultured in confocal dishes and incubated with a 2-μM BODIPY-FA mix for 15 minutes. Cells were then fixed with 4% paraformaldehyde for 30 minutes, and the nuclei were counterstained with 4′,6-diamidino-2-phenylindole (DAPI; 4083, Cell Signaling Technology, Boston, MA, USA). Following fixation, cells were washed three times with pre-chilled PBS. Images were acquired with an FV1000 confocal laser scanning microscope (CLSM).

### Animal model construction

Three different mouse models of CRC were studied. The experiments were approved by the Animal Ethics Committee of Southern Medical University (L2015006) and conducted in accordance with the animal experimentation guidelines of the Chinese Ministry of Health. For the generation of the chemically induced mouse model, six- to eight-week-old male Balb/c mice were intraperitoneally injected with 10 mg/kg body weight azoxymethane (AOM; A5486, Sigma-Aldrich, Saint Louis, MO, USA). One week later, 2.5% dextran sulfate sodium (DSS; MW 40000–50000 Da, MP, Santa Ana, California, USA) was dissolved in distilled water and administered for seven days, followed by 14 days of normal drinking water [27]. This cycle was repeated three times, and when the last DSS cycle ended, the mice were prepared for *in vivo* staining. Our run-in phase experiments demonstrated that the neoplastic regions always occurred near the rectum, with increased thickness and stiffness of the bowel wall macroscopically; these observations guided the topical application of the fluorescent agents. We successfully acquired 32 AOM/DSS-induced neoplastic mice; 14 were used for the first fluorescence microscopy imaging study (six for BODIPY-FA; six for 2-Deoxy-2-[(7-nitro-2,1,3-benzoxadiazol-4-yl)amino]-D-glucose (2-NBDG); and two for acriflavine hydrochloride), and 18 were used for *in vivo* CLE imaging (eight for BODIPY-FA; seven for 2-NBDG; and three for acriflavine hydrochloride).

For the xenograft model, 10^6^ human LOVO CRC cells were orthotopically implanted through a laparotomy into the colonic serosa layer of Balb/c null mice (Medical Experimental Animal Centre of Guangdong Province, China) under stringent aseptic conditions. Subsequently, the abdominal cavity was closed with a commonly used disinfectant. Three to four weeks after the implantation, the mice received a second laparotomy for the next step. In total, 16 xenograft mice were successfully acquired, including eight for fluorescence microscopy imaging and eight for *in vivo* CLE imaging (four for BODIPY-FA staining and four for 2-NBDG staining).

APC^-min^ mice (purchased from the Animal Model Research Centre of Nanjing University, China) are a widely accepted model of intestinal tumorigenesis. Six APC^-min^ mice (four to six months old) received follow-up BODIPY-FA imaging.

### Staining in the mouse models

Prior to the application of fluorescent agents, AOM/DSS-induced mice, xenograft mice and APC^-min^ mice were fasted for 12 h. Anesthesia was administered with 0.01 g/mL pentobarbital, given as 6–7 μL/g body weight. For *in vivo* neoplastic staining, a 200 μM BODIPY-FA mix, 500 μM 2-NBDG (N13195, Invitrogen) and 0.05% acriflavine (A8251, Sigma-Aldrich) were prepared, and the colons and/or small intestines of the mice were isolated through laparotomy after a 2–3-cm long segment of the tract was created with two vascular hemoclips. Then, 100 μL of the fluorescent agent was injected directly into the lumen. After five minutes of staining, the stained areas were exposed, and the unbound BODIPY-FA was rinsed off three times with PBS.

### Patients and tissue specimens

The sporadic colorectal neoplasm patients came from the Departments of Gastroenterology and General Surgery, Nanfang Hospital, Guangzhou, China. These patients were selected based on the following factors: (1) complete clinical follow-up data were available, and (2) the patients had not eaten large amounts of high-fat food or been taking lipid medicine. The Institutional Research Medical Ethics Committee of Nanfang Hospital granted approval for the present study (NFEC-2015-083). Informed written consent was obtained from patients before examination.

### RT-PCR

Total RNA was extracted with TRIzol (Takara) from paired normal and cancerous tissues from CRC patients, and cDNA was synthesized with TAKARA reverse transcriptase (037A, TAKARA, China) with oligo-dT primers. The cDNA and SYBR Green Premix ExTaq were used for subsequent real-time PCR amplification on an LC480 System. The primer sequences are listed in Supplementary Table 5.

### Western blotting

The protein levels of fatty acid synthase (FASN), fatty acid binding protein 1 (FABP1), Caveolin-1 and CD36 in the paired human CRC samples were examined by Western blotting. Antibodies for FASN, FABP1, Caveolin-1, CD36 and GAPDH were purchased from Cell Signaling Technology. Protein blots were incubated with the indicated primary antibodies and then with the appropriate secondary antibodies, and detection was performed with enhanced chemiluminescence according to the manufacturer's instructions.

### Histochemistry

Active specimens from each region of the mouse models were immediately embedded with Optimum Cutting Temperature compound in liquid nitrogen for cryosectioning after the staining procedure. After being counterstained with DAPI, the cryosections were observed with fluorescence microscopy. The other cryosections were stained with H&E. Finally, the fluorescence images were matched with their corresponding H&E images. The mean fluorescence intensity was calculated for statistical analysis. H&E images were analyzed independently by B.N. and H.Z.

### CLE system

CLE (EC3830FK, Pentax, Japan) integrates experimental confocal microscopy with conventional optical endoscopy, delivering an excitation wavelength of 488 nm and an emission wavelength of 505–585 nm. A confocal probe was placed onto the mucosal layer for scanning, and optical sections of 475 × 475 μm were obtained. The axial resolution was 7 μm, and the lateral resolution was 0.7 μm (1024 × 1024 pixels). The imaging depth (z-axis) varied from 0–250 μm below the surface layer in 4-μm increments. Confocal images were obtained at a fixed laser power of 500 μW and standardized brightness.

### Staining of human tissues and CLE imaging

Patients were randomly divided into the fluorescein sodium group and the BODIPY-FA group (detailed in Supplementary Tables 1.1 and 1.2). Fluorescein sodium (5–10 mL of a 10% solution, Baiyunshan Mingxing Pharmaceutical Co.) was administered intravenously according to a published protocol [28]. The 200-μM BODIPY-FA mix was applied topically for five minutes and then rinsed off with PBS.

**Table 1:** Confocal laser endomicroscopy criteria for BODIPY-FA imaging for colorectal pathology

Grade	Criteria
Normal	Round luminal openings Regular arrangement and distribution of crypts A homogeneous layer of epithelial cells with normal polarity Interspersed goblet cells Approximately 10–15 crypts per field of view
Hyperplasia	Star-shaped crypt openingsFocal aggregation of regularly shaped crypts Morphologically normal epithelial cells with cellular polarity Normal or reduced number of goblet cells Inflammatory infiltrates in the lamina propria, reduced number of crypts
Low-grade IN	Elongated or branch-like crypts, preserved crypt integritySlightly irregular arrangement of crypts with no crypt fusion Basally localized nuclei, single layer of nuclei Reduced number of goblet cells Nucleus-to-cytoplasm ratio < 1:1 Slightly darker images than the adjacent normal images
High-grade IN	A complete loss of normal tissue arrangement, with multiple irregular crypts and lateral fusion Ridged, lined or extremely branched crypt structures Elevated crypt/stroma ratio and dramatically reduced number of goblet cells Nucleus-to-cytoplasm ratio > 1:1 Significantly darker images than the adjacent normal images
Well-differentiated carcinoma	Loss of crypts and cellular orientation while gland structures are still visible Basement membrane is broken but still visible Disorganized, enlarged nucleus that occupies most of the cell volume Total loss of goblet cells Significantly darker images than the normal sites from the same patient
Poorly-differentiated carcinoma	Mixed and dispersed cancer cells without gland formationSignificantly darker images than the normal regions of the same patient

For CLE imaging, when the confocal window is fully placed on the surface mucosa (for both *in vivo* mouse models and human tissues) and the imaging plane depth is real-time adjusted during ongoing screening, the observation time for each site should be shorter than 20 s. CLE was performed by two experienced endomicroscopists (W.G. or T.L.).

### Confocal pattern classification

For the assessment of confocal images, clear images of each site were selected and evaluated according to previous classifications [20, 29] by two different endoscopy doctors, with three main histopathologic characteristics taken into consideration. Disagreements between the two observers were resolved by discussion, and the final diagnosis was agreed upon by both physicians. One characteristic was crypt morphology, which allowed the images to be classified into normal mucosa, hyperplastic locations and neoplasia (intraepithelial neoplasia [IN] and adenocarcinoma). The second characteristic was nuclear visualization, which permitted differentiation among low-grade IN, high-grade IN and adenocarcinoma. The last characteristic was the apparent darkness of the neoplasm compared with normal areas of the same patient under the same conditions. A detailed classification of the BODIPY-FA images from our patient samples is shown in Table 1. The H&E biopsy images were judged independently by two experts and graded according to the modified Vienna classification [30]. In addition, clear images with intravenous fluorescein sodium were selected and judged in strict accordance with previous classification criteria [20].

### Statistical analysis and evaluation

Mean greyscale values and mean fluorescence intensities were analyzed with Image J (NIH, Bethesda, MD, USA). CLE images were analyzed based on the following standards: (1) the observation time was less than 20 s per site and (2) recognizable cellular structures were visible. ROIs of 100 × 100 μm from three different regions per image were selected for the calculation of the mean greyscale value and mean fluorescence intensity. Data were analyzed with the statistical software package SPSS v13.0 (SPSS Inc., Chicago, IL, USA). Significance was determined with Student's *t*-test, one-way or two-way ANOVA, or a nonparametric test, as appropriate. The Kolmogorov-Smirnov test was performed to compare relative protein expression in the primary data of 95 tumor samples from the Clinical Proteomic Tumor Analysis Consortium. All *P* values were generated with two-sided tests, and *P <* 0.05 was defined as significant.

### Double-blind trial for CLE diagnosis

Seventy-one target locations from 17 patients, including up to 508 confocal images (mean: 7 per site, range: 5–20) with BODIPY-FA, and an additional 68 target locations from 45 patients, including up to 950 images (mean: 14 per site, range: 7–65) with fluorescein sodium, were randomly coded and separately sent to two expert endomicroscopists (FC.Z. and F.H.D.) who were blinded to the macroscopic appearance and H&E diagnosis. All data were analyzed with SAS 9.4 software. The overall validity was calculated to compare the predictive ability of confocal endomicroscopy with that of the gold-standard H&E diagnosis. The Bhapkar test and Kappa coefficient were used to evaluate the diagnostic difference and consistency, respectively, between H&E staining and CLE with either BODIPY-FA or fluorescein sodium [31]. The Kappa coefficient was also used to evaluate the inter-observer agreement of the CLE diagnoses with BODIPY-FA or fluorescein sodium staining.

### Writing assistance

We acknowledge Dr. Zhifei Shao at Stanford University for his help in editing this manuscript.

## SUPPLEMENTARY MATERIALS FIGURES AND TABLES


