# Supplementary material for: Use of confocal laser endomicroscopy with a fluorescently labeled fatty acid to diagnose colorectal neoplasms
Source: Oncotarget. 2017 Jul 24;8(35):58934–47. doi: 10.18632/oncotarget.19515 (PMC5601704; doi:10.18632/oncotarget.19515)
Supplement: Supplementary file 1 [file oncotarget-08-58934-s001.pdf]

Use of confocal laser endomicroscopy with a fluorescently labeled fatty acid to diagnose colorectal neoplasms

SUPPLEMENTARY MATERIALS

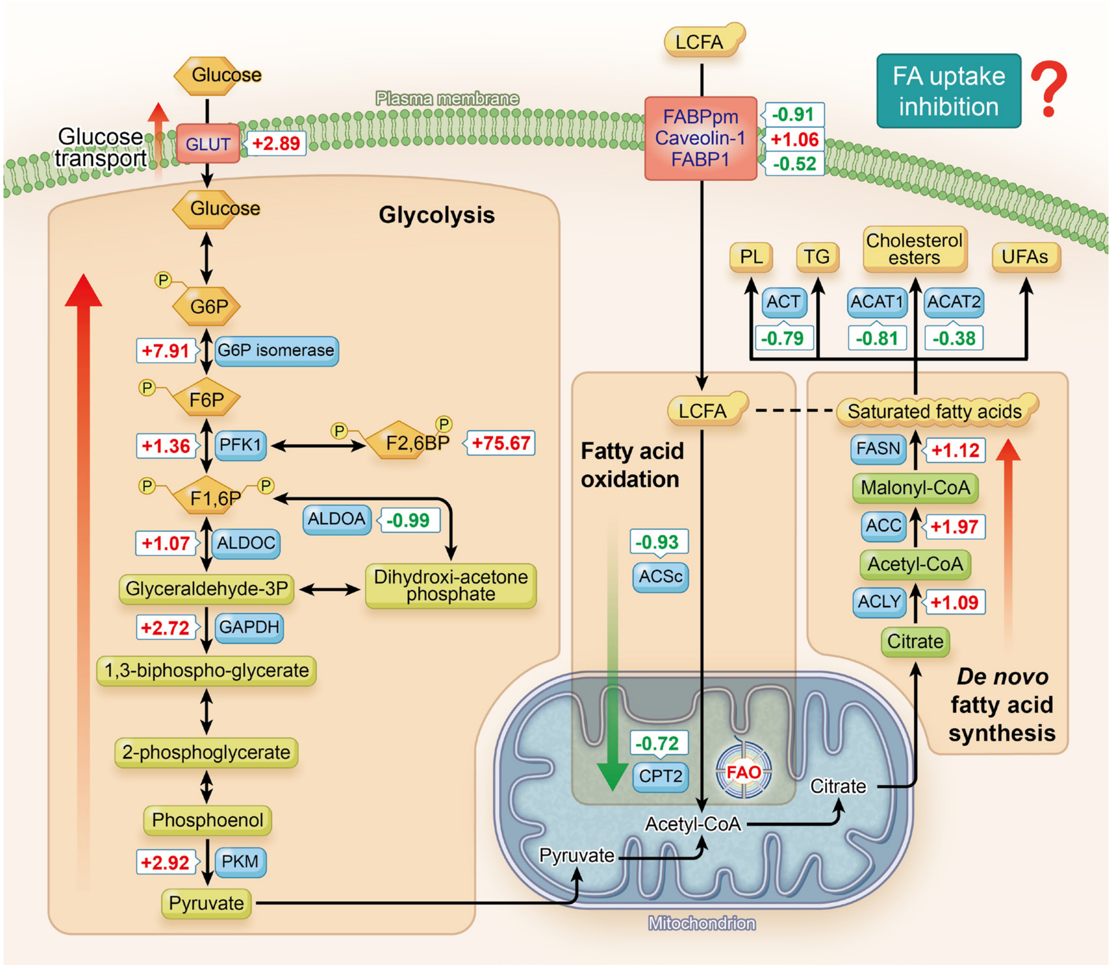

Supplementary Figure 1: Elevated *de novo* fatty acid synthesis with reduced fatty acid oxidation from the primary data of 95 CRC samples with mass spectrometry-based proteomics (from the Clinical Proteomic Tumor Analysis Consortium). The numbers beside the enzymes represent the fold-changes of the protein levels in cancerous samples versus matched normal samples. Red text indicates increased expression, whereas green text indicates reduced expression of the proteins in the cancerous samples. LCFA, long-chain fatty acid; GLUT, glucose transporter; G6P, glucose-6-phosphate; PFK1, phosphofructokinase1; ALDOA(C), aldolase A(C); GAPDH, glyceraldehyde-3-phosphate dehydrogenase; PKM, pyruvate kinase; ACLY, ATP citrate lyase; ACC, acetyl-CoA carboxylase; FASN, fatty acid synthase; ACSc, acetyl-CoA synthetase complex; CPT, carnitine palmitoyl transferase; FAO, fatty acid oxidation; PL, phospholipids; TG, triglycerides; UFAs, unsaturated fatty acids; ACT, acyl-CoA transferase; ACAT, acyl coenzyme A-cholesterol acyltransferase.

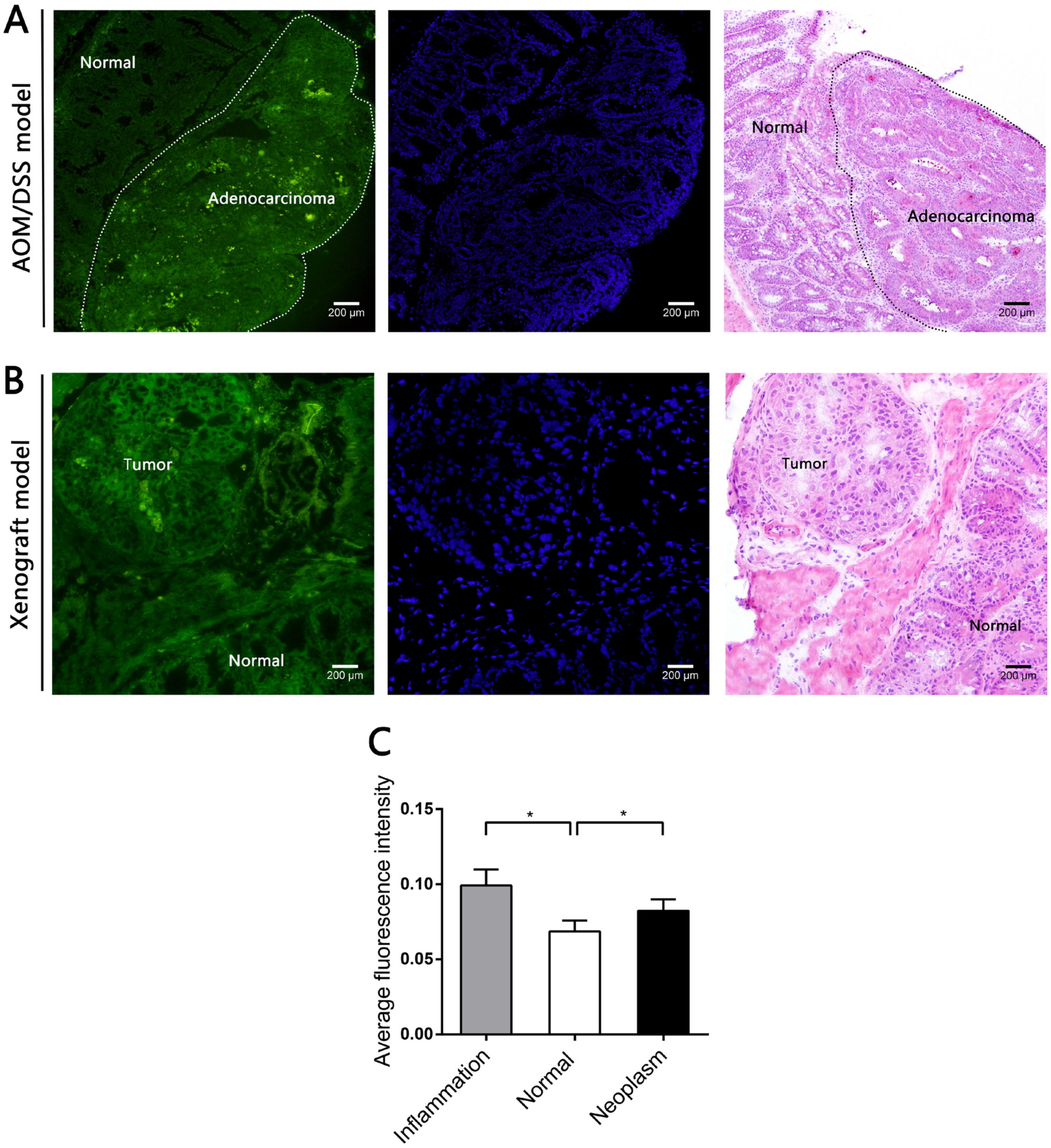

Supplementary Figure 2: Fluorescence microscopy imaging after topical application of 2-NBDG to the mucosae of mouse models, and the corresponding DAPI nuclear counterstaining and H&E images. (**A**) 2-NBDG signal at the transition between normal and adenocarcinoma mucosae in an AOM/DSS-induced mouse. Images were obtained at 100×. (**B**) 2-NBDG staining of a LOVO xenograft mouse at the transition between normal and tumorous tissues, four weeks after implantation, at 200×. (**C**) The average fluorescence intensity of 2-NBDG in different tissue types of AOM/DSS-induced mice. $^*P < 0.05$, $^{**}P < 0.01$. Bars indicate the SEMs.

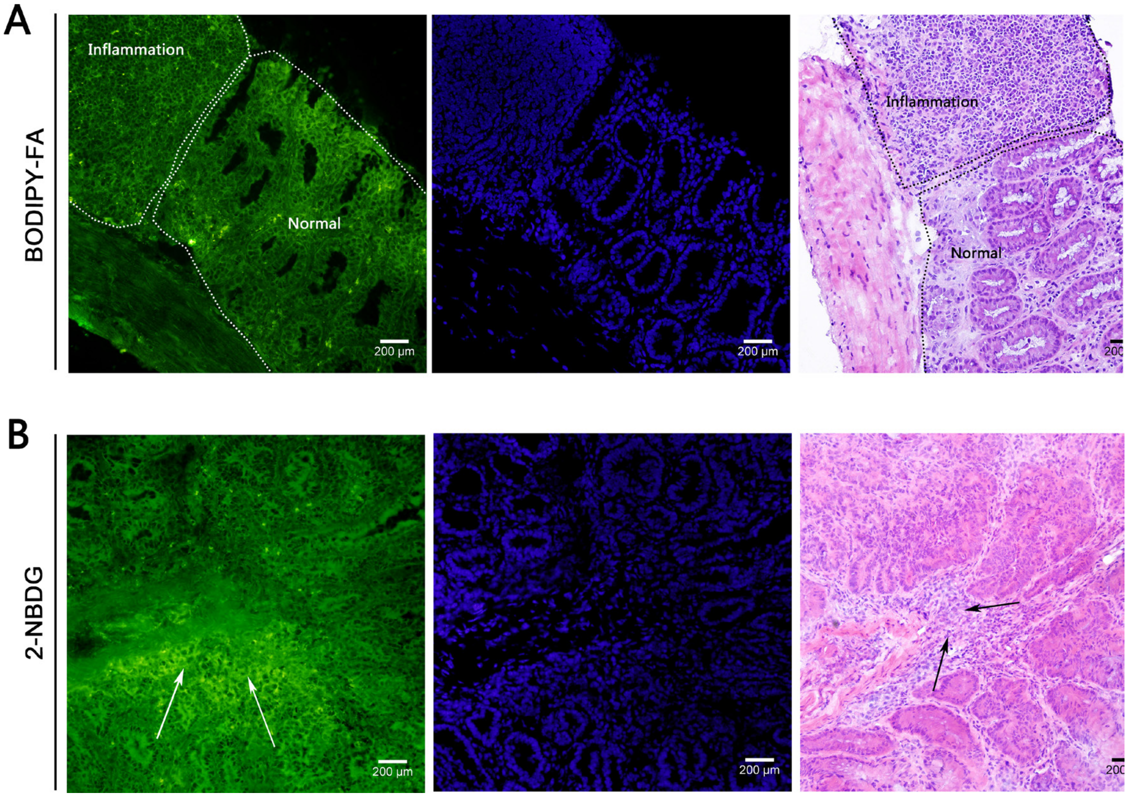

Supplementary Figure 3: Fluorescence microscopy imaging of inflammation after topical application of fluorescent agents to the mucosae of AOM/DSS mice, and the corresponding DAPI nuclear counterstaining and H&E images, at 200×. (**A**) BODIPY-FA staining at the transition between normal and inflamed regions. (**B**) 2-NBDG staining at the transition between adenocarcinoma and inflammation. Arrows point to inflammatory sites in the neoplastic background.

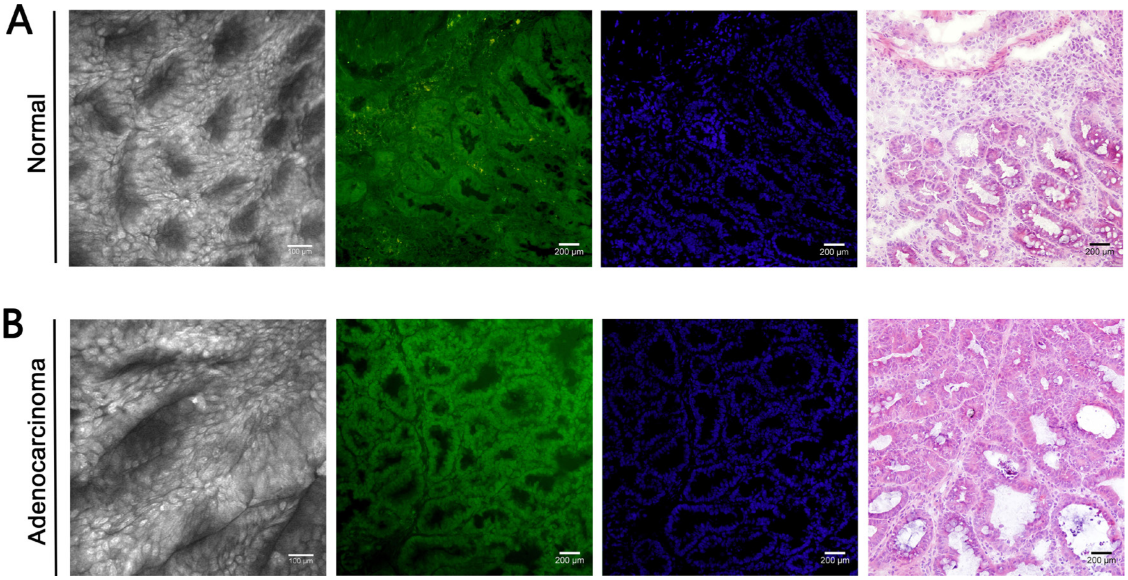

Supplementary Figure 4: Nonspecific acriflavine imaging of AOM/DSS-induced mice with CLE, and corresponding fluorescent and H&E images. (**A**) Imaging of normal mucosa. (**B**) Imaging of adenocarcinoma mucosa. All nuclei were clearly observed.

Supplementary Table 1.1: Details of Patients with BODIPY-FA staining and CLE involved

No.	Age	Gender	Location	Selected examination sites	Histopathological diagnosis
1	66	Female	Sigmoid	2	Normal/Hyperplasia
2	66	Female	Rectum	8	Normal/Well-differentiated adenocarcinoma
3	38	Female	Sigmoid	3	Poorly-differentiated adenocarcinoma
4	79	Male	Rectum	5	Well/Moderately-differentiated adenocarcinoma
5	59	Male	Colon	0	Bad imaging
6	57	Female	Sigmoid	7	Normal/Hyperplasia/Well-differentiated adenocarcinoma
7	46	Male	Rectum	7	Hyperplasia/Well-differentiated adenocarcinoma
8	42	Female	Rectum	1	Well/Moderately-differentiated adenocarcinoma
9	53	Female	Sigmoid	0	Bad imaging
10	33	Male	Sigmoid	9	Normal/Hyperplasia/High-grade IN
11	59	Male	Rectum	7	Normal/High-grade IN
12	56	Female	Sigmoid	3	High-grade IN
13	53	Female	Rectum	3	Low-grade IN
14	25	Female	Rectum	3	Low-grade IN
15	48	Male	Ascending colon	1	Low-grade IN
16	35	Male	Rectum	6	Normal / Hyperplasia / Low-grade IN
17	65	Male	Colon	6	Hyperplasia / Low-grade IN

71 specimens with histologically proven normal mucosae (n = 8), hyperplasia (n = 8), CIN (n = 33: 14 with low-grade IN and 19 with high-grade IN), or adenocarcinomas (n = 22: 19 with well/moderately-differentiated carcinoma and 3 with poorly-differentiated carcinoma).

Supplementary Table 1.2: Details of patients with intravenous fluorescein sodium involved

No.	Age	Gender	Location	Selected examination sites	Histopathological diagnosis
1	54	Male	Colon	3	Normal/High-grade IN
2	69	Female	Sigmoid	1	Low-grade IN
3	56	Female	Sigmoid	1	Low-grade IN
4	77	Female	Sigmoid	1	High-grade IN
5	51	Male	Sigmoid	1	Low-grade IN
6	50	Male	Sigmoid	3	Low-grade IN
7	53	Female	Anus	1	High-grade IN
8	39	Female	Rectum	1	Well-differentiated adenocarcinoma
9	54	Male	Sigmoid	1	Low-grade IN
10	65	Male	Sigmoid	2	Well-differentiated adenocarcinoma
11	68	Female	Sigmoid	2	Low-grade IN/Well-differentiated adenocarcinoma
12	58	Male	Rectum	2	Low-grade IN/Well-differentiated adenocarcinoma
13	72	Female	Rectum	1	Well-differentiated adenocarcinoma
14	48	Male	Colon	1	Low-grade IN
15	72	Male	Colon	1	Low-grade IN
16	55	Male	Rectum	1	Low-grade IN
17	64	Male	Rectum	1	Well-differentiated adenocarcinoma
18	32	Male	Sigmoid	1	Low-grade IN
19	86	Male	Rectum	2	Normal/Well-differentiated adenocarcinoma
20	50	Female	Colon	2	Low-grade IN
21	48	Female	Colon	3	Normal/High-grade IN
22	59	Male	Rectum	3	Well-differentiated adenocarcinoma
23	54	Male	Rectum	3	Hyperplasia
24	39	Male	Rectum	2	High-grade IN
25	60	Male	Sigmoid	1	High-grade IN
26	42	Female	Rectum	2	Well-differentiated adenocarcinoma
27	63	Male	Anus	1	Well-differentiated adenocarcinoma
28	52	Male	Sigmoid	3	Hyperplasia
29	47	Male	Sigmoid	1	Low-grade IN
30	26	Male	Colon	1	Low-grade IN
31	71	Male	Sigmoid	1	Low-grade IN
32	45	Male	Rectum	3	Hyperplasia
33	46	Male	Rectum	1	Hyperplasia
34	58	Male	Colon	1	Low-grade IN
35	39	Male	Rectum	1	High-grade IN
36	50	Male	Rectum	1	Hyperplasia
37	45	Male	Sigmoid	1	Low-grade IN
38	62	Female	Colon	2	Normal/Low-grade IN
39	70	Female	Sigmoid	1	Low-grade IN
40	64	Male	Rectum	1	Low-grade IN
41	39	Female	Rectum	1	Low-grade IN
42	34	Male	Sigmoid	1	Low-grade IN
43	67	Female	Rectum	1	Low-grade IN
44	57	Female	Colon	1	Low-grade IN
45	53	Male	Rectum	2	Poorly-differentiated adenocarcinoma

68 specimens with histologically proven normal mucosae (n = 8), hyperplasia (n = 9), CIN (n = 35: 26 with low-grade IN and 9 with high-grade IN), or adenocarcinomas (n = 16: 14 with well-differentiated carcinoma and 2 with poorly-differentiated carcinomas).

Supplementary Table 1.3: Details of CRC patients' samples for RT-PCR, western blotting and isolation of primary colonic epithelial cells

Variable	All cases
**Gender**	
Male	22
Female	20
**Age (years)**	
≤ 60	19
> 60	23
**Location**	
Colon	5
Sigmoid	17
Rectum	20
**Histopathological diagnosis**	
Well/moderately-differentiated adenocarcinoma	34
Poorly-differentiated adenocarcinoma	8

Supplementary Table 2: Correlation between confocal diagnosis and colorectal pathology

H&E	CLE													
	BODIPY-FA							Fluorescein sodium						
	**N**	**−**	$+_1$	$+_2$	$+_3$	$+_4$	**Total**	**N**	**−**	$+_1$	$+_2$	$+_3$	$+_4$	**Total**
**N**	8	0	0	0	0	0	8	8	0	0	0	0	0	8
**−**	0	8	0	0	0	0	8	0	4	5	0	0	0	9
$+_1$	0	4	10	0	0	0	14	0	5	14	7	0	0	25
$+_2$	0	0	5	12	2	0	19	0	0	4	1	4	0	9
$+_3$	0	0	0	2	14	3	19	0	0	1	0	10	3	14
$+_4$	0	0	0	0	2	1	3	0	0	0	0	1	1	2
**Total**	8	12	15	14	18	4	71	8	9	24	8	15	4	68

N, Normal mucosa; -, Hyperplasia; $+_1$, Mucosal low-grade IN; $+_2$, High-grade IN; $+_3$, Well-differentiated adenocarcinoma; $+_4$, Poorly-differentiated adenocarcinoma; H&E, hematoxylin and eosin.

Supplementary Table 3.1: Inter-observer agreement for CLE with BODIPY-FA staining

Observer 2	Observer 1						
	N	−	$+_1$	$+_2$	$+_3$	$+_4$	**Total**
N	8	1	0	0	0	0	9
−	0	10	1	0	0	0	11
$+_1$	0	0	13	3	2	0	18
$+_2$	0	0	0	9	1	0	10
$+_3$	0	0	0	4	12	3	19
$+_4$	0	0	0	0	1	3	4
**Total**	8	11	14	16	16	6	71

Supplementary Table 3.2: Inter-observer agreement for CLE with fluorescein sodium staining

Observer 2	Observer 1						
	N	−	$+_1$	$+_2$	$+_3$	$+_4$	**Total**
N	7	0	0	0	0	0	7
−	1	6	6	3	1	0	17
$+_1$	0	0	14	4	0	0	18
$+_2$	0	0	4	2	1	0	7
$+_3$	0	0	1	0	14	0	15
$+_4$	0	0	0	0	0	4	4
**Total**	8	6	25	9	16	4	68

N, Normal mucosa; -, Hyperplasia; $+_1$, Mucosal low-grade IN; $+_2$, High-grade IN; $+_3$, Well-differentiated adenocarcinoma; $+_4$, Poorly-differentiated adenocarcinoma.

Supplementary Table 4: Details of relative expression of enzymes involved in glucose and lipid metabolism from the clinical proteomic tumor analysis consortium data

PROTEIN NAME	PROTEIN ID	GENE NAME	N	S.D.	C	S.D.	C/N	KS-test
F2,6BP	O60825	PFKFB2	0.032934	0.022223	2.492253	0.226572	75.674440	4.58E-18
G6P	P06744	GPI	0.830929	0.152033	6.574184	0.059388	7.911846	3.63E-21
GAPDH	P04406	GAPDH	3.103492	0.147134	8.447361	0.043996	2.721889	3.63E-21
ACC	Q13085	ACACA	0.862107	0.131738	1.695654	0.157323	1.966871	9.92E-05
PFK1	Q01813	PFKP	3.304898	0.069234	4.495385	0.118768	1.360219	2.09E-16
FASN	P49327	FASN	6.011456	0.107732	6.710358	0.150985	1.116262	1.31E-05
ACLY	P53396	ACLY	4.7165	0.078595	5.148689	0.066446	1.091633	1.31E-05
ALDOC	P09972	ALDOC	5.209076	0.033877	5.553933	0.061010	1.066203	3.59E-08
Caveolin-1	Q03135	CAV1	2.368266	0.08637	2.5005	0.214716	1.055836	0.12
ALDOA	P04075	ALDOA	7.473005	0.018613	7.389457	0.050394	0.988820	0.0009
ACSc	Q86V21	AACS	0.960851	0.12436	0.891477	0.133695	0.927799	0.05
FABPpm	P00505	GOT2	6.072722	0.02281	5.504391	0.072733	0.906412	2.89E-12
CPT2	P23786	CPT2	5.19023	0.044616	3.758026	0.115919	0.724058	1.71E-18
FABPL	P07148	FABP1	7.598407	0.059237	3.950924	0.295323	0.519967	3.63E-21
PKM	P14618	PKM	2.655357	0.010291	7.756315	0.003711	2.921006	3.63E-21
GLUT	Q9BYW1	SLC2A11	0.794258	0.018368	2.294125	0.011315	2.888388	2.89E-12
ACAT1	P24752	ACAT1	6.100740	0.006980	4.912245	0.006172	0.805188	3.81E-18
ACAT2	O75908	ACAT2	3.683965	0.013598	1.385962	0.011901	0.376215	3.94E-18
ACT	P55809	OXCT1	3.653298	0.006980	2.893801	0.010355	0.792107	3.59E-08

C, cancer; N, normal; S.D., standard deviation; KS, Kolmogorov-Smirnov.

Supplememntary Table 5: Specific primer sequences for quantitative real-time PCR

hFATP4-Forward	CGGTTCTGGGACGATTGTAT
hFATP4-Reverse	AACCTGGTGCTGGTTTTCTG
hCaveolin-1-Forward	CCGCGACCCTAAACACCTC
hCaveolin-1-Reverse	GCCTTCCAAATGCCGTCAA
hFABPpm-Forward	AACTAGCCCTGGGTGAGAAC
hFABPpm-Reverse	CCTGTGAAGTCAAAACCGCA
hCD36-Forward	GGGAAAGTCACTGCGACATG
hCD36-Reverse	TGCAATACCTGGCTTTTCTCA
hFABP1-Forward	CTGACCTCTGGCCGCTATTC
hFABP1-Reverse	TCTTCCGGCAGACCGATTG
